# To Investigate the Potential Mechanism of Huanglian Jiangtang Formula Lowering Blood Sugar in View of Network Pharmacology and Molecular Docking Technology

**DOI:** 10.1155/2023/2827938

**Published:** 2023-02-16

**Authors:** Fang xu Li, Ziting Zhou, Cheng Lu, Guoming Pang, Zhigang Lu

**Affiliations:** ^1^Hospital of Traditional Chinese Medicine, Kaifeng 475000, Henan, China; ^2^Nanjing University of Traditional Chinese Medicine, Nanjing 210023, Jiangsu, China; ^3^Institute of Basic Research in Clinical Medicine, China Academy of Chinese Medical Sciences, Beijing 100700, China

## Abstract

**Objective:**

In view of network pharmacology and molecular docking technology, to explore the targets as well as effect mechanism of the Huanglian Jiangtang formula (including *Coptis chinensis*, *Anemarrhena asphodeloides*, rhubarb wine, *Cortex Moutan*, *Rehmannia glutinosa*, and *dried ginger*) in the type II diabetes therapy.

**Methods:**

TCMSP and Batman database (DB) were used to retrieve the chemical components and action targets of drugs; GeneCards, OMIM, TTD, DrugBank, and other databases were applied to screen the disease targets. We used the UniProt DB to annotate the targets before building the drug-compound-target network with Cytoscape 3.9.1. We also exploited the String DB to construct the protein-protein interaction (PPI) network. In addition, the targets for the treatment of type II diabetes were searched in the DrugBank, OMIM, GeneCards, and TTD database; then, we utilized Venn to intersect the key targets for the therapy of type II diabetes and active ingredient targets to obtain common targets. Furthermore, we exploited the common targets using GO and KEGG enrichment analysis method. The common targets and core components were analyzed by molecular docking using the AutoDock software.

**Results:**

A total of 61 effective components of this compound were screened out; drugs and type II diabetes have 278 common targets; the PPI network screened core target proteins such as CDKN1A, CDK2, and E2F1 with the help of molecular docking technology; the three main compounds including quercetin, kaempferol, and gamma-aminobutyric acid were obtained. Besides, the key target proteins had excellent binding properties with the main components. The signal pathways of six compound interventions in type II diabetes were mostly related to cancer, cocaine addiction, aminoacyl-tRNA biosynthesis, glycine, serine, threonine metabolism, platinum drug resistance, and other pathways, according to the KEGG enrichment analysis method.

**Conclusion:**

In the treatment of diabetes, the Huanglian Jiangtang formula has sorts of properties especially in the aspects of composition, target, and pathway. Its molecular target and mechanism of action may be related to pathways in cancer, cocaine addiction, aminoacyl-tRNA biosynthesis, glycine, serine, threonine metabolism, platinum drug resistance, and other pathways. This conclusion can provide theoretical support and science for further research.

## 1. Introduction

Diabetes mellitus (DM) is a typical endocrine and metabolic disorders marked by high blood glucose levels. As a common and frequently occurring disease in modern lifestyles, DM is also a refractory disease requiring lifelong treatment. Although it will not cause severe reactions in a short time, serious complications will be induced if it is not treated in time. Diabetes complications include retinopathy, nephropathy, and peripheral neuropathy [1].

Due to its extremely complex etiology and pathogenesis, it has not been fully clarified so far. Clinical treatment is generally based on lifestyle interventions such as diet and exercise, and hypoglycemic drugs are used for treatment. For example, chlorpropamide and clofibrate can treat diabetes insipidus complicated with diabetes [2]. Insulin therapy is also widely used in diabetes. Treatment for type II diabetes mellitus may become an earlier start of the insulin therapy to preserve the remaining pancreatic insulin reserve. Furthermore, the combination of thiazolidinediones and insulin in the therapies of type II diabetes is effective [3].

In addition, research has demonstrated that bone therapy structural diagnosis and bone therapy manipulative treatment have superior efficacy in the management of diabetes and the comorbidities associated with the condition [4]. Because DM is a chronic disease, the comprehensive treatment of traditional Chinese medicine has great superiorities. As a result of increased investigation into the mechanisms behind the development of diabetes DM, the mechanism of hypoglycemic drugs has also been enriched. At present, research shows that the traditional Chinese medicine treatment of DM is mainly from stimulating insulin secretion, protecting and repairing islets of Langerhans-*β* Cells, improving insulin resistance, inhibiting glucosidase activity, inhibiting glucose absorption in the body, promoting glucose utilization, improving the level of oxidative stress, and other ways to play a hypoglycemic role. The promotion of the traditional Chinese medicine as a therapy for diabetes relies on the findings of research on individual traditional Chinese medicines that have been shown to have a hypoglycemic impact [5].

Emerging network pharmacology has been used in recent years to screen drug molecular action targets using computer simulation and various databases. It also predicts the signal pathway and action mechanism of traditional Chinese medicine in the treatment of diabetes, and it carries out experimental verification when it is required. It is derived from system biology as its foundation and uses computer simulation and various databases to screen drug molecular action targets, to explain the process of disease development, and through signal pathway analysis, and it possesses the qualities of “multigenes, multitargets, multiple pathways, and integrated regulation,” all of which are congruent with the dialectical style of thinking that is utilized in the traditional Chinese medicine. For instance, Zhang et al. used molecular docking and network pharmacology to identify Cinnamomi Ramulus, *Paeonia lactiflora* Pall, and *Anemarrhenae Rhizoma* Decoction in order to explore the effective components and important ways to treat diabetes. They came to the conclusion that the combination of molecular docking and network pharmacology is a feasible strategy to explore the bioactive components and mechanisms of traditional Chinese medicine. At the same time, they clearly understood that Cinnamomi Ramulus, *Paeonia lactiflora* Pall, and *Anemarrhenae Rhizoma* Decoction are all able to successfully treating diabetes through the use of multiple elements, multiple targets, and many approaches [6].

**Figure 1:**
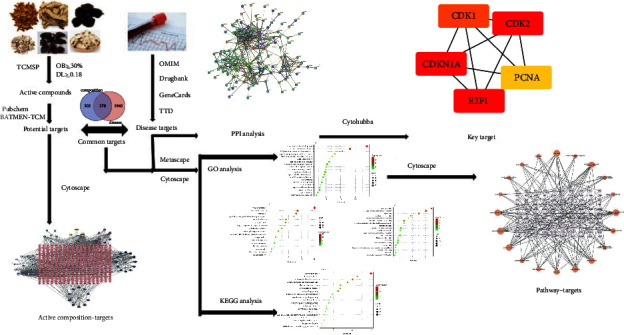
The active constituents and probable targets of six drugs (including HL, ZM, JDH, DP, SDH, and GJ) in the compound were predicted from different databases. From four separate databases, pertinent diabetic markers were gathered. Then, we use Venn to find the intersection of active substance targets and disease target, which is approved to be common targets. According to the network analysis of the prevalent target bioactive components, the essential active compounds were discovered. In order to find overlapping paths, it was necessary to examine the enrichment of disease targets and overlap targets. The data we analyzed through go and KEGG are made into corresponding bubble charts through the map website. Finally, the coherence between targets of key important chemical substances as well as key pathways was analyzed to explore the hypoglycemic mechanism of the compound.

Accordingly, to explore the hypoglycemic mechanism of the compound of *Coptis chinensis* (HL), *Anemarrhena asphodeloides* (ZM), wine-processed rhubarb (JDH), Cortex Moutan (DP), *Rehmannia glutinosa* (SDH), and dried ginger (GJ), we explored the target and pathway of hypoglycemic effect of this compound according to the network pharmacology and molecular docking technologies. The flow chart of our result is presented in Figure 1.

## 2. Materials and Methods

### 2.1. Applied Database

TCMSP (Traditional Chinese Medicine Systems Pharmacology) (https://tcmspw.com/tcmsp.php); PubChem (https://pubchem.ncbi.nl-m.nih.gov/); BATMAN-TCM; Swiss Target Prediction (https://www.swisstargetprediction.ch/); Uniprot (https://www.uniprot.org/); OMIM (https://mirror.omim.org/); DrugBank; GeneCards (https://www.genecards.org/); TTD-DatabaseCommons (https://ngdc.cncb.ac.cn/databasecommons/database/id/4948); String (https://cn.string-db.org/); Metascape (https://metascape.org/gp/index.html#/main/step1); Cytoscape (version 3.9.1); PDB (https://www.rcsb.org/); PyMOL were used.

### 2.2. Active Ingredients in Compound

We filtered the active components of the compound throughout TCMSP using the keywords “Huanglian,” “Zhimu,” “Danpi,” and “Ganjiang,” with the requirements OB ≥ 30% and DL ≥ 0.18. Jiuda Huang and Sheng Di Huang's structural details and molecular makeup were gleaned from PubChem and BATMAN-TCM, respectively.

### 2.3. Objectives Associated with Active Ingredients

Targets for the compound's active components were taken from the TCMSP DB. Due to the possibility that the targets offered by TCMSP may be lacking, we additionally looked for the targets on similarity-based target prediction websites called BATMEN-TCM and Swiss Target Prediction, to predict the targets of “Jiangda Huang” and “Sheng Di Huang” in the compound. An Internet bioinformatics analytical tool called BATMEN-TCM is used to investigate the molecular basis of TCM. BATMEN-TCM's parameters are adjusted to score ≥20 and fixed *p* ≤ 0.05. An online program called the Swiss Target Prediction attempts to forecast molecular targets. The species is limited to “*Homo sapiens*” in the Swiss Target Prediction. By screening in UniProt and building the network diagram of the “traditional Chinese medicine compound target,” all the retrieved genes were finally standardized.

### 2.4. Targets Related to Diabetes

DrugBank, OMIM, GeneCards, and TTD were consulted for targets that were pertinent to DM. Using “Diabetes” as the keyword, we found 2218 targets from each of these databases that are related to diabetes. In UniProt DB, these targets were likewise standardized.

### 2.5. Common Targets of Compound and Diabetes

The intersection of the illness target, as well as active chemical targets, is then discovered using Venn. The common genes obtained after the intersection are the relevant targets of the compound that may act on DM.

### 2.6. PPI Network Construction

The String is a database of known and predicted protein-protein interactions. A database of observed and anticipated protein-protein interactions is called The String. It presently has 24.6 million proteins from 5,090 different organisms. Potential target interactions were examined in the current study utilizing String DB with the organism setting set to “*Homo sapiens*” and a confidence level of less than 0.4. Using Cytoscape, a common targets PPI network was created and shown. The CytoHubba was used to identify hub genes; moreover, the top ten genes produced by the greatest neighborhood component called an MCC technique were considered as hub genes. Thus, we upload the targets obtained under item 1.5 to the String DB, set the type as “*Homo sapiens*,” hide free nodes, build the PPI network diagram, and further analyze and optimize the PPI network diagram with Cytoscape 3.9.1. An online network topology investigation was carried out using the network analyzer plug-in. The degree value, which indicates the number of connections with other nodes and shows the importance of a node, is a crucial metric to evaluate for each node in an interactive network. Then, the key targets are obtained through analysis.

### 2.7. GO and KEGG Pathway Enrichment Analysis

Metascape was used to analyze the enrichment of go and KEGG pathways, and the results were visualized through a bubble diagram. More genes are represented by a larger bubble, and the higher the importance of enrichment is indicated by a darker hue. In order to identify and clarify the roles of transcription factors from three perspectives, namely, biological processes (BPs), cell components (CCs), and molecular functions (MFs), annotation is used in gene ontology (GO). Besides, information about the genome, biochemistry, and normal functioning is combined in the database known as KEGG. Accordingly, we uploaded the abbreviation of the intersection target obtained in the previous step to the David database in gene symbol format for go enrichment analysis, and the variation is statistically meaningful, according to KEGG enrichment analysis (*p* < 0.05). Through in-depth analysis of go enrichment, this study discusses the biological process, molecular function, cellular components, and other potential targets of the compound bioactive components in the treatment of DM; KEGG enrichment analysis was used to discover the pathway distribution of potential targets of the compound active ingredients in the treatment of DM.

### 2.8. Molecular Docking

Micromolecules are docked into biological macromolecules during the molecular docking technique in order to score their equivalent beliefs at the binding sites. A crucial step in the framework drug design process is docking micromolecule compounds into the linker of a receptor and calculating the binding interactions of the complex. Both a precise and rapid docking process and the capacity to observe binding geometries and interactions are essential for a complete comprehension of the basic rules that govern the strength of a protein/ligand combination. Therefore, we utilized molecular docking technology including PDB debates, PubChem, PyMol, and AutoDock tools software to verify the binding ability of key target proteins with chemical active ingredients.

## 3. Results

### 3.1. The Principal Active Components

A total of 61 active components were found in the compound using TCMSP DB with the standards of OB of more than 30% and DL of more than 0.18, of which 14 were from *Coptis chinensis* (HL), 15 from *Anemarrhena asphodeloides* (ZM), 16 from wine-processed rhubarb (JDH), 11 were from Cortex Moutan (DP), and 5 were from dried ginger (GJ). In Supplementary Table 1, specific information on HL, ZM, JDH, DP, and GJ is provided. The target prediction webservers described above (BATMEN-TCM and Swiss Target Prediction) anticipated the targets of *Rehmannia glutinosa* (SDH). Detailed information on SDH is listed in Supplementary Table 2.

### 3.2. Targets Related to Active Ingredients

**Figure 2:**
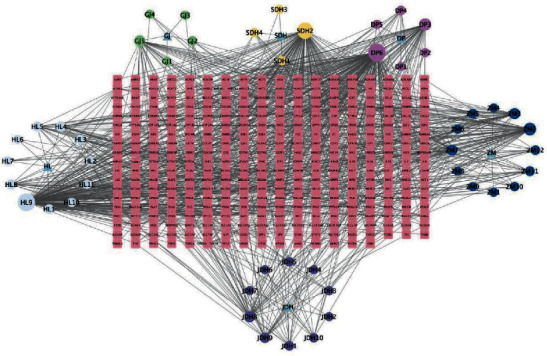
Traditional Chinese medicine-compound-target network diagram.

By using targets prediction function in the TCMSP DB, corresponding target protein was found through data screening. Then, the duplicate and invalid values were deleted, and 339 compound action targets were obtained. Introduce the compound drugs, drug active ingredients, and targets into Cytoscape 3.9.1 and build the relationship network diagram of compound chemical active ingredients and predicted targets, as shown in Figure 2 (the target, symbolized by the square, is pictured inside the circle, which represents the active substance).

### 3.3. Common Targets

**Figure 3:**
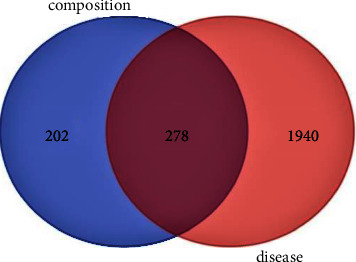
Venn diagram of drug prediction target and disease target.

Through the disease target database under item 1.1, the weight of the targets obtained from each database was removed, and finally, 2218 DM-related targets were obtained. Match with the targets of the effective ingredients of the compound, construct the “drug disease” targets Venn diagram, and get a total of 278 common targets to provide relevant data for subsequent PPI and GO enrichment analysis, as shown in Figure 3.

### 3.4. Construction of PPI Networks

**Figure 4:**
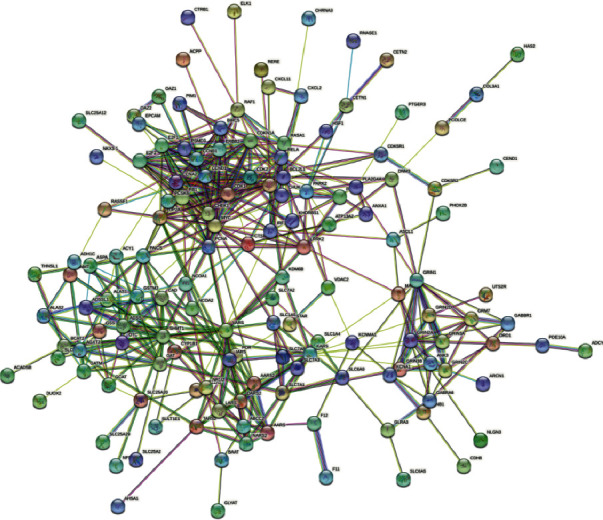
PPI network.

**Figure 5:**
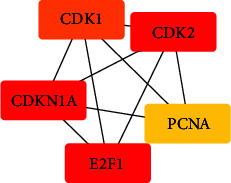
Key targets.

Utilizing String database DB, enter the intersection gene of compound prescription and DM to acquire the PPI network as depicted in Figure 4, import obtained data into Cytoscape 3.9.1, and use the MCC algorithm in CytoHubba plug-in to analyze the data to evaluate out just the top 10 genes, which are key targets. It can be seen from Figure 5 that the core genes CDKN1A, CDK2, E2F1, CDK1, and PCNA in the top 5 of the degree value are analyzed. Wang et al. have confirmed that the above five genes are closely related to DM, providing a strong basis for the next experiment [7–9].

### 3.5. GO Biological Process Enrichment Analysis

**Figure 6:**
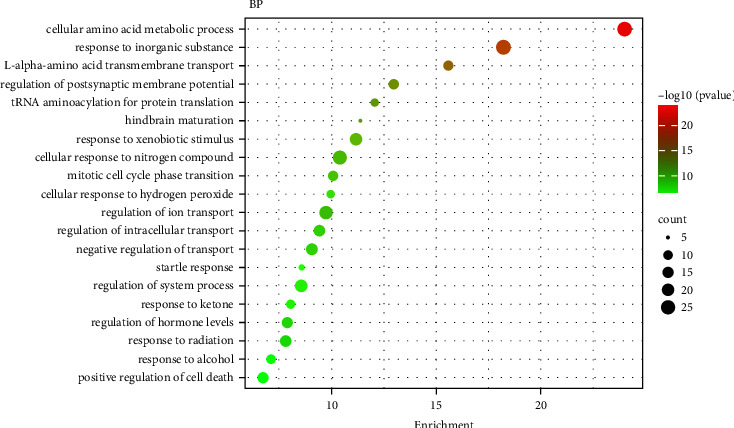
Top 20 GO analysis of biological processes.

**Figure 7:**
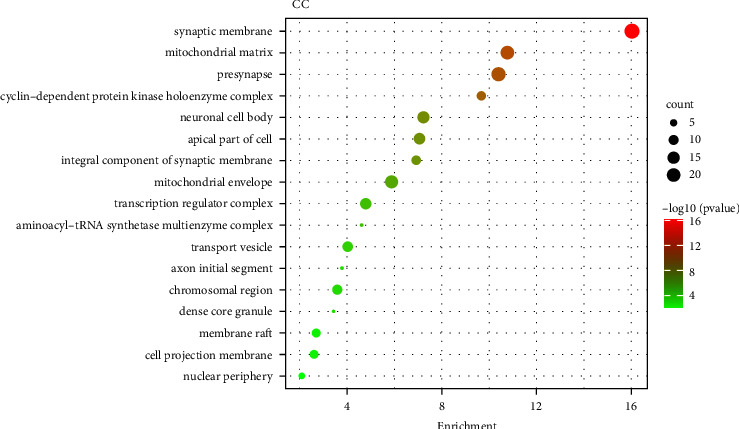
Top 20 GO analysis of cellular components.

**Figure 8:**
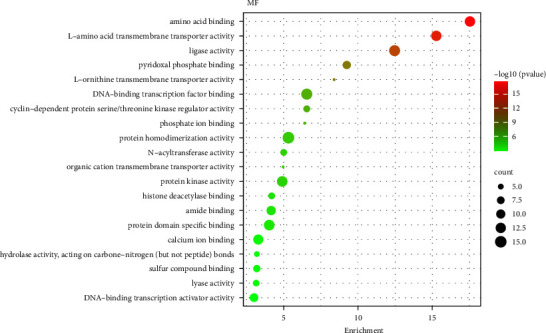
Top 20 GO analysis of molecular functions.

To obtain the biological effects of key target proteins, GO biological function enrichment analysis was carried out on the intersection targets. It was found that the main targets were enriched on 969 entries, including 706 BP, 174 CC, and 89 MF. BP showed that it may be related to biological processes such as cellular amino acid metabolic process and response to inorganic substance L and alpha-amino acid transmembrane transport. Shopit et al. have researched that DM is closely related to the above processes [10]. The results of CC showed that it may be closely related to the synaptic membrane, mitochondrial matrix, and presynapse. Meng et al. have researched that DM is closely related to the synaptic membrane [11]. The results of MF showed that it may be related to amino acid binding, L-amino acid transmembrane transporter activity, ligase activity, and so on. The results of the top 20 move analysis for BP, CC, and MF are shown in Figures 6–8, correspondingly.

### 3.6. KEGG Pathway Enrichment Analysis

**Figure 9:**
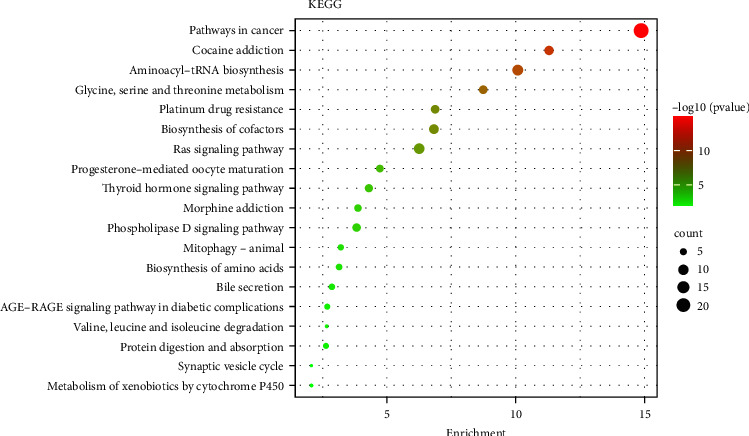
Top 10 KEGG pathway.

**Figure 10:**
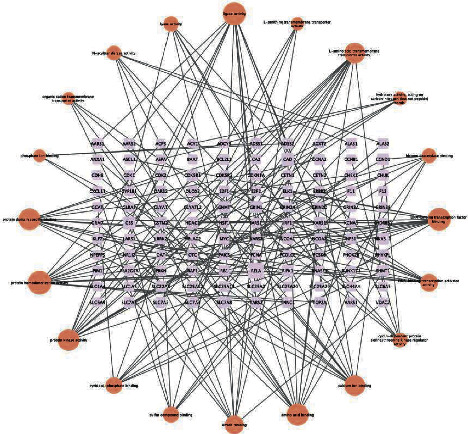
Pathway-key target network.

The purpose of the KEGG pathway analysis is to obtain the pathways enriched by the key targets and carry them out on the key targets. According to the findings, the most important targets of compound therapy for DM were enriched on 104 pathways, including pathways in cancer, cocaine addiction, aminoacyl-tRNA biosynthesis, glycine, serine, threonine metabolism, platinum drug resistance, and other pathways. The top ten results of the KEGG enrichment study are represented by bubbles in Figure 9, which is a representation of the results. Among them, CDK2, E2F1, CDKN1A, CDK1, CCND1, and CCNB1, which are located in the core genes in the PPI network, involve multiple pathways in the top ten, indicating that CDK2, E2F1, CDKN1A, CDK1, CCND1, and CCNB1 may be the key targets of compound therapy for DM. Finally, the pathway-key target network is built through Cytoscape 3.9.1, as shown in Figure 10.

### 3.7. Molecular Docking Results of Active Ingredients and Key Targets

**Figure 11:**
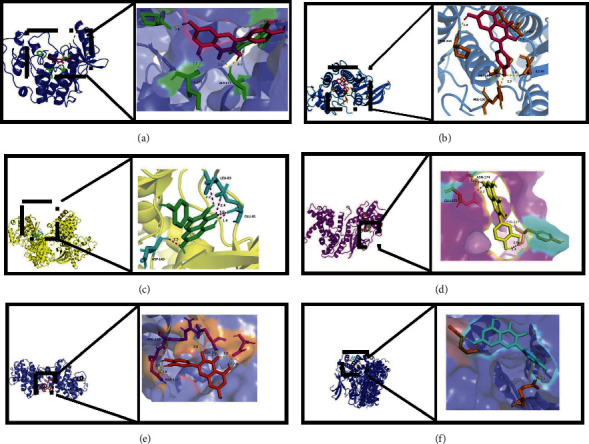
Binding mode of target protein and compound. (a) Quercetin and CDK2. (b) Kaempferol and CDK2. (c) Kaempferol and E2F1. (d) Kaempferol and CCND1. (e) Quercetin and CCNB1. (f) Kaempferol and CCNA2.

From this analysis, the 6 target proteins with the highest importance values obtained in the PPI network were docked. The purpose of this docking was to investigate whether the primary bioactive constituents of the compound played a role in hypoglycemia through the compound's key targets, which were CDK2, E2F1, CDKN1A, CDK1, and CCND1 and CCNB1. Aiming at obtaining the crystal structure of key targets from the database, we downloaded PDB format files and used AutoDock 4.2.6 software to dock the compounds and target proteins one by one (as shown in Table 3). It can be seen from Supplementary Table 3 that the docking energy between the small molecule compounds contained in the compound and the key target protein is smaller than 0 kcal·mol^−1^, indicating that the binding is excellent. With the help of PyMol 2.5.0 software, the molecular docking visualization of gamma-aminobutyric acid, quercetin, kaempferol, CDK2, E2F1, and CCND1 was carried out in Figure 11.

## 4. Discussion

In view of the research method of network pharmacology, this work studies the hypoglycemic mechanism of the compound active ingredients composed of HL, ZM, JDH, SDH, DP, and GJ; the results showed that the compound had 54 active compounds, as well as 2218 type 2 DM targets, were found. The two summarized and obtained 278 common hypoglycemic targets of the compound composed of *Coptis chinensis*, *Anemarrhena asphodeloides*, Rhubarb, Cortex Moutan, *Rehmannia glutinosa*, and dried ginger. Protein interaction network analysis and KEGG enrichment analysis showed that the functional proteins corresponding to the five genes CDK2, E2F1, CDKN1A, CDK1, and CCND1 and CCNB1 may be the key proteins for the compound to play the role of hypoglycemic; the KEGG enrichment analysis obtained seven signal pathways: protein domain specific binding, protein homodimerization activity, protein kinase activity, DNA-binding transcription factor binding, ligase activity, cyclin-dependent protein serineL-amino acid transmembrane transporter activity, calcium ion binding, and amino acid binding. At present, the compound drug researched by Quazi et al. has proved that the extract possesses numerous classes of chemicals such as alkaloids, glycosides, tannins, polyphenols, and terpenoids, which can contribute to the antidiabetic activity through alpha-amylase inhibition [12, 13].

Traditional Chinese medicine is commonly used to treat diabetes, and the studies have shown that some traditional Chinese medicine has a better effect on reducing blood glucose, such as Coptis polysaccharide, and other substances in *Coptis chinensis* have a significant synergistic effect on reducing blood glucose [14]. Furthermore, studies have shown that andrographolide has a strong antidiabetic effect and has hypoglycemic and hypolipidemic effects on type II diabetic rates [15]. It is the active part of *Anemarrhena asphodeloides* to reduce blood glucose.

There are obvious differences between network pharmacology and traditional pharmacology [16]. The biggest distinguishing point is that network pharmacology studies the regulatory effect of drugs on biomolecular networks from the direction of the whole body and the whole system. Because systematicity, correlation, and predictability are the most prominent features, the network pharmacology is an essential method for analyzing the efficacious ingredients of traditional Chinese medicine and for treating disease targets. There are many ways to treat DM [17], such as insulin injection, oral hypoglycemic drugs, surgical treatment, and so on, but they cannot be completely cured. Different drugs can also inhibit the rise of blood glucose through a variety of regulatory pathways. For example, insulin secretagogues, biguanides, insulin sensitizers, alpha-glucosidase inhibitors, incretin mimetics, amylin antagonists, and sodium-glucosecotransporter-2 (SGLT2) inhibitors [18]. It can be inferred that there are some coordination hubs in the mechanism pathway of drug hypoglycemia.

However, if we only use network pharmacology to explore a certain mechanism of drugs, it will inevitably be biased and limited, because several omissions cannot be ignored [19]. First, there are differences between the components in different patients and those in theoretical research, which will affect the operation process of the mechanism pathway [20]. Second, it is difficult for network pharmacology to effectively distinguish the inhibition and activation between monomer components when discussing drug effects, and there will also be unavoidable deviations in the process of GO and KEGG enrichment analysis. On the other hand, the thinking construction of network pharmacology depends on the database, but at the current stage, the database information required for network pharmacology is not very comprehensive, and the data of CO participation examination and query are limited. Under this condition, research on drugs based on network pharmacology will seriously affect the authenticity of experimental results. This study preliminarily reveals the pathway of the hypoglycemic mechanism of the compound composed of HL, ZM, JDH, SDH, DP, GJ, which provides a reference basis for further experimental research and clinical application of the compound. However, there are some limitations in the network pharmacology method, and more pathways of its hypoglycemic mechanism need to be further experimentally researched to complete a more comprehensive supplement. Therefore, our study continues to explore the method of molecular docking to explore the exact location where the main active components of this compound played a hypoglycemic role through the key targets.

## Data Availability

The active ingredients in the compound data used to support the findings of this study are included within the supplementary information file.

## Supplementary Materials

Supplementary Materials The specific information on HL, ZM, JDH, DP, and GJ is provided in Supplementary Table 1; some detailed information on SDH is listed in Supplementary Table 2; and the docking energy between the small molecule compounds contained in the compound and the key target protein is shown in Supplementary Table 3.
