# Supplementary material for: To Investigate the Potential Mechanism of Huanglian Jiangtang Formula Lowering Blood Sugar in View of Network Pharmacology and Molecular Docking Technology
Source: Evid Based Complement Alternat Med. 2023 Feb 16;2023:2827938. doi: 10.1155/2023/2827938 (PMC9950321; doi:10.1155/2023/2827938)
Supplement: Supplementary Materials — The specific information on HL, ZM, JDH, DP, and GJ is provided in Supplementary Table 1; some detailed information on SDH is listed in Supplementary Table 2; and the docking energy between the small molecule compounds contained in the compound and the key target protein is shown in Supplementary Table 3. [file 2827938.f1.zip › Table 1.pdf]

Table 1:Main chemical components of HL,ZM,JDH,DP,GJ

Mol ID	Molecule Name	OB (%)	DL
MOL001454	berberine	36.86	0.78
MOL013352	Obacunone	43.29	0.77
MOL002894	berberrubine	35.74	0.73
MOL002897	epiberberine	43.09	0.78
MOL002903	(R)-Canadine	55.37	0.77
MOL002904	Berlambine	36.68	0.82
MOL002907	Corchoroside A_qt	104.95	0.78
MOL000622	Magnograndiolide	63.71	0.19
MOL000762	Palmidin A	35.36	0.65
MOL000785	palmatine	64.6	0.65

MOL000098	quercetin	46.43	0.28
MOL001458	coptisine	30.67	0.86
MOL002668	Worenine	45.83	0.87
MOL008647	Moupinamide	86.71	0.26
**Mol ID**	**Molecule Name**	**OB (%)**	**DL**
MOL001677	asperglaucide	58.02	0.52
MOL003773	Mangiferolic acid	36.16	0.84
MOL000422	kaempferol	41.88	0.24
MOL004373	Anhydroicaritin	45.41	0.44
MOL004489	Anemarsaponin F_qt	60.06	0.79
MOL004492	Chrysanthemaxanthin	38.72	0.58

MOL004497	Hippeastrine	51.65	0.62
MOL004514	Timosaponin B III_qt	35.26	0.87
MOL000449	Stigmasterol	43.83	0.76
MOL004528	Icariin I	41.58	0.61
MOL004540	Anemarsaponin C_qt	35.5	0.87
MOL004542	Anemarsaponin E_qt	30.67	0.86
MOL000483	(Z)-3-(4-hydroxy-3-methoxy-phenyl)-N-[2-(4-hydroxyphenyl)ethyl]acrylamide	118.35	0.26
MOL000546	diosgenin	80.88	0.81
MOL000631	coumaroyltyramine	112.9	0.2
**Mol ID**	**Molecule Name**	**OB (%)**	**DL**
MOL002235	EUPATIN	50.8	0.41
MOL002251	Mutatochrome	48.64	0.61

MOL002259	Physciondiglucoside	41.65	0.63
MOL002260	Procyanidin B-5,3'-O-gallate	31.99	0.32
MOL002268	rhein	47.07	0.28
MOL002276	Sennoside E_qt	50.69	0.61
MOL002280	Torachrysone-8-O-beta-D-(6'-oxayl)-glucoside	43.02	0.74
MOL002281	Toralactone	46.46	0.24
MOL002288	Emodin-1-O-beta-D-glucopyranoside	44.81	0.8
MOL002293	Sennoside D_qt	61.06	0.61
MOL002297	Daucosterol_qt	35.89	0.7
MOL002303	palmidin A	32.45	0.65
MOL000358	beta-sitosterol	36.91	0.75

MOL000471	aloe-emodin	83.38	0.24
MOL000554	gallic acid-3-O-(6'-O-galloyl)-glucoside	30.25	0.67
MOL000096	(-)-catechin	49.68	0.24
**Mol ID**	**Molecule Name**	**OB (%)**	**DL**
MOL001925	paeoniflorin_qt	68.18	0.4
MOL000211	Mairin	55.38	0.78
MOL000359	sitosterol	36.91	0.75
MOL000422	kaempferol	41.88	0.24
MOL000492	(+)-catechin	54.83	0.24
MOL007003	benzoyl paeoniflorin	31.14	0.54
MOL007369	4-O-methylpaeoniflorin_qt	67.24	0.43

MOL007374	5-[[5-(4-methoxyphenyl)-2-furyl]methylene]barbituric acid	43.44	0.3
MOL007382	mudanpioside-h_qt 2	42.36	0.37
MOL007384	paeonidanin_qt	65.31	0.35
MOL000098	quercetin	46.43	0.28
**Mol ID**	**Molecule Name**	**OB (%)**	**DL**
MOL002514	Sexangularetin	62.86	0.3
MOL002501	[(1S)-3-[(E)-but-2-enyl]-2-methyl-4-oxo-1-cyclopent-2-enyl] (1R,3R)-3-[(E)-3-methoxy-2-methyl-3-oxoprop-1-enyl]-2,2-dimethylcyclopropane-1-carboxylate	62.52	0.31
MOL002464	1-Monolinolein	37.18	0.3
MOL000359	sitosterol	36.91	0.75
MOL000358	beta-sitosterol	36.91	0.75
