# Supplementary material for: To Investigate the Potential Mechanism of Huanglian Jiangtang Formula Lowering Blood Sugar in View of Network Pharmacology and Molecular Docking Technology
Source: Evid Based Complement Alternat Med. 2023 Feb 16;2023:2827938. doi: 10.1155/2023/2827938 (PMC9950321; doi:10.1155/2023/2827938)
Supplement: Supplementary Materials — The specific information on HL, ZM, JDH, DP, and GJ is provided in Supplementary Table 1; some detailed information on SDH is listed in Supplementary Table 2; and the docking energy between the small molecule compounds contained in the compound and the key target protein is shown in Supplementary Table 3. [file 2827938.f1.zip › Table 2.pdf]

Table 2:Main chemical components and target of JDH

ID	Compound	Predicted targets
SDH1	Campesterol	ESR1
SDH1	Campesterol	PGR
SDH1	Campesterol	AR
SDH1	Campesterol	NR3C1
SDH1	Campesterol	ANXA1
SDH2	Gamma-Aminobutyric Acid	CACNA2D1
SDH2	Gamma-Aminobutyric Acid	GRIN2A
SDH2	Gamma-Aminobutyric Acid	GRIN3A
SDH2	Gamma-Aminobutyric Acid	RIPK1
SDH2	Gamma-Aminobutyric Acid	SLC25A20
SDH2	Gamma-Aminobutyric Acid	LCMT1
SDH2	Gamma-Aminobutyric Acid	CPT2
SDH2	Gamma-Aminobutyric Acid	ACADSB
SDH2	Gamma-Aminobutyric Acid	IARS
SDH2	Gamma-Aminobutyric Acid	SLC1A5
SDH2	Gamma-Aminobutyric Acid	SHMT1
SDH2	Gamma-Aminobutyric Acid	SLC7A8
SDH2	Gamma-Aminobutyric Acid	ALAS1
SDH2	Gamma-Aminobutyric Acid	SLC1A4
SDH2	Gamma-Aminobutyric Acid	GLRA3

SDH2	Gamma-Aminobutyric Acid	GLYAT
SDH2	Gamma-Aminobutyric Acid	SLC25A12
SDH2	Gamma-Aminobutyric Acid	NARS2
SDH2	Gamma-Aminobutyric Acid	VDAC2
SDH2	Gamma-Aminobutyric Acid	GPT
SDH2	Gamma-Aminobutyric Acid	SLC6A9
SDH2	Gamma-Aminobutyric Acid	OAZ1
SDH2	Gamma-Aminobutyric Acid	ADSS
SDH2	Gamma-Aminobutyric Acid	CAD
SDH2	Gamma-Aminobutyric Acid	GABBR1
SDH2	Gamma-Aminobutyric Acid	LYZ
SDH2	Gamma-Aminobutyric Acid	CDK5R2
SDH2	Gamma-Aminobutyric Acid	UTS2
SDH2	Gamma-Aminobutyric Acid	EPCAM
SDH2	Gamma-Aminobutyric Acid	FASLG
SDH2	Gamma-Aminobutyric Acid	CETN1
SDH2	Gamma-Aminobutyric Acid	CYP11A1
SDH2	Gamma-Aminobutyric Acid	SLC44A4
SDH2	Gamma-Aminobutyric Acid	DNM3
SDH2	Gamma-Aminobutyric Acid	UTS2R
SDH2	Gamma-Aminobutyric Acid	UROD
SDH2	Gamma-Aminobutyric Acid	PHOX2B

SDH2	Gamma-Aminobutyric Acid	ADCY3
SDH2	Gamma-Aminobutyric Acid	LRRK2
SDH2	Gamma-Aminobutyric Acid	KDM6B
SDH2	Gamma-Aminobutyric Acid	PCSK6
SDH2	Gamma-Aminobutyric Acid	PLAT
SDH2	Gamma-Aminobutyric Acid	PLG
SDH2	Gamma-Aminobutyric Acid	GRIN2D
SDH2	Gamma-Aminobutyric Acid	ASCL1
SDH2	Gamma-Aminobutyric Acid	IARS2
SDH2	Gamma-Aminobutyric Acid	THNSL1
SDH2	Gamma-Aminobutyric Acid	LARS
SDH2	Gamma-Aminobutyric Acid	CPT1A
SDH2	Gamma-Aminobutyric Acid	SLC22A5
SDH2	Gamma-Aminobutyric Acid	GOT2
SDH2	Gamma-Aminobutyric Acid	ABAT
SDH2	Gamma-Aminobutyric Acid	PPAT
SDH2	Gamma-Aminobutyric Acid	GCAT
SDH2	Gamma-Aminobutyric Acid	ADSSL1
SDH2	Gamma-Aminobutyric Acid	DARS2
SDH2	Gamma-Aminobutyric Acid	AARS2
SDH2	Gamma-Aminobutyric Acid	PHYKPL
SDH2	Gamma-Aminobutyric Acid	TNNC1

SDH2	Gamma-Aminobutyric Acid	OAZ2
SDH2	Gamma-Aminobutyric Acid	AARS
SDH2	Gamma-Aminobutyric Acid	SLC7A3
SDH2	Gamma-Aminobutyric Acid	KARS
SDH2	Gamma-Aminobutyric Acid	SLC7A1
SDH2	Gamma-Aminobutyric Acid	OTC
SDH2	Gamma-Aminobutyric Acid	ACY1
SDH2	Gamma-Aminobutyric Acid	PAICS
SDH2	Gamma-Aminobutyric Acid	PDE5A
SDH2	Gamma-Aminobutyric Acid	KHDRBS1
SDH2	Gamma-Aminobutyric Acid	F12
SDH2	Gamma-Aminobutyric Acid	COMT
SDH2	Gamma-Aminobutyric Acid	TH
SDH2	Gamma-Aminobutyric Acid	KCNB1
SDH2	Gamma-Aminobutyric Acid	GHRL
SDH2	Gamma-Aminobutyric Acid	CHRNA3
SDH2	Gamma-Aminobutyric Acid	HMGCR
SDH2	Gamma-Aminobutyric Acid	INS
SDH2	Gamma-Aminobutyric Acid	STAR
SDH2	Gamma-Aminobutyric Acid	CDH8
SDH2	Gamma-Aminobutyric Acid	TACR2
SDH2	Gamma-Aminobutyric Acid	F11

SDH2	Gamma-Aminobutyric Acid	GRM7
SDH2	Gamma-Aminobutyric Acid	GRIN3B
SDH2	Gamma-Aminobutyric Acid	GRIN2C
SDH2	Gamma-Aminobutyric Acid	GRIN1
SDH2	Gamma-Aminobutyric Acid	ADA
SDH2	Gamma-Aminobutyric Acid	VARS
SDH2	Gamma-Aminobutyric Acid	SLC25A29
SDH2	Gamma-Aminobutyric Acid	TARS2
SDH2	Gamma-Aminobutyric Acid	XDH
SDH2	Gamma-Aminobutyric Acid	BCAT2
SDH2	Gamma-Aminobutyric Acid	BAAT
SDH2	Gamma-Aminobutyric Acid	SLC7A2
SDH2	Gamma-Aminobutyric Acid	ASPA
SDH2	Gamma-Aminobutyric Acid	NFS1
SDH2	Gamma-Aminobutyric Acid	GSS
SDH2	Gamma-Aminobutyric Acid	GNMT
SDH2	Gamma-Aminobutyric Acid	GPR18
SDH2	Gamma-Aminobutyric Acid	GATM
SDH2	Gamma-Aminobutyric Acid	OAT
SDH2	Gamma-Aminobutyric Acid	SLC25A2
SDH2	Gamma-Aminobutyric Acid	ALAS2
SDH2	Gamma-Aminobutyric Acid	GLYATL2

SDH2	Gamma-Aminobutyric Acid	RNASE1
SDH2	Gamma-Aminobutyric Acid	SLC6A5
SDH2	Gamma-Aminobutyric Acid	AGXT2
SDH2	Gamma-Aminobutyric Acid	SLC25A13
SDH2	Gamma-Aminobutyric Acid	ABCC2
SDH2	Gamma-Aminobutyric Acid	DGKI
SDH2	Gamma-Aminobutyric Acid	PTEN
SDH2	Gamma-Aminobutyric Acid	ARCN1
SDH2	Gamma-Aminobutyric Acid	CETN2
SDH2	Gamma-Aminobutyric Acid	KCNA1
SDH2	Gamma-Aminobutyric Acid	PRSS12
SDH2	Gamma-Aminobutyric Acid	CEND1
SDH2	Gamma-Aminobutyric Acid	CDK5R1
SDH2	Gamma-Aminobutyric Acid	ANK3
SDH2	Gamma-Aminobutyric Acid	DRD4
SDH2	Gamma-Aminobutyric Acid	ATP13A2
SDH2	Gamma-Aminobutyric Acid	SPX
SDH2	Gamma-Aminobutyric Acid	RERE
SDH2	Gamma-Aminobutyric Acid	PARK2
SDH2	Gamma-Aminobutyric Acid	NLGN3
SDH3	Catalpol	POLA1
SDH4	Rehmaglutin C	HDAC2

SDH4	Rehmaglutin C	PRKCA
SDH4	Rehmaglutin C	PRKCD
SDH4	Rehmaglutin C	HMGCR
SDH4	Rehmaglutin C	ITGB2
SDH4	Rehmaglutin C	ITGAL
