# Supplementary material for: To Investigate the Potential Mechanism of Huanglian Jiangtang Formula Lowering Blood Sugar in View of Network Pharmacology and Molecular Docking Technology
Source: Evid Based Complement Alternat Med. 2023 Feb 16;2023:2827938. doi: 10.1155/2023/2827938 (PMC9950321; doi:10.1155/2023/2827938)
Supplement: Supplementary Materials — The specific information on HL, ZM, JDH, DP, and GJ is provided in Supplementary Table 1; some detailed information on SDH is listed in Supplementary Table 2; and the docking energy between the small molecule compounds contained in the compound and the key target protein is shown in Supplementary Table 3. [file 2827938.f1.zip › Table 3.pdf]

Table3:Binding Energy

Binding Energy (kcal • mol^{-1})						
targets / active ingredient	CDK2	E2F1	CDKN1A	CCNB1	CCND1	CCNA2
gamma aminobutyric acid	-3.65	-3.13	-2.64	-2.47	-2.64	-2.56
quercetin	-5.71	-4.62	-3.75	-5.0	-3.86	-4.35
kaempferol	-5.6	-5.34	-4.74	-4.94	-5.6	-4.91
